# Introducing a Series of Reviews Assessing Engagement in Evidence Syntheses

**DOI:** 10.1002/cesm.70057

**Published:** 2025-11-20

**Authors:** Jennifer Petkovic, Joanne Khabsa, Lyubov Lytvyn, Alex Todhunter‐Brown, Olivia Magwood, Pauline Campbell, Elie A. Akl, Thomas W. Concannon, Holger Schunemann, Vivian Welch, Peter Tugwell

**Affiliations:** ^1^ University of Ottawa Ottawa Ontario Canada; ^2^ Clinical Research Institute American University of Beirut Beirut Lebanon; ^3^ Department of Health Research Methods, Evidence, and Impact (HEI) McMaster University Hamilton Canada; ^4^ Glasgow Caledonian University Glasgow UK; ^5^ Bruyere Health Research Institute Ottawa Canada; ^6^ Department of Nursing and Community Health Glasgow Caledonian University Glasgow UK; ^7^ Department of Internal Medicine American University of Beirut Beirut Lebanon; ^8^ The RAND Corporation and Tufts University School of Medicine Boston Massachusetts USA; ^9^ Clinical Epidemiology and Research Center (CERC) Humanitas University & Humanitas Research Hospital Milan Italy; ^10^ Department of Medicine University of Ottawa Ottawa Ontario Canada

## 1 Introduction

High quality evidence syntheses are used in health decision‐making, such as policies, legislation, and clinical recommendations [1]. The usefulness, relevance, meaningfulness, and accessibility of evidence syntheses may be improved when people who are affected by those decisions, called “interest‐holders,” are included in the evidence synthesis process [2, 3, 4]. This concept of engagement in research is based on the principle that those affected by the health condition under study or the intervention to address it have a moral right to contribute to the decisions about how the research is conducted [3, 5]. While there are increasing expectations from funders regarding the involvement of interest‐holders [6], the most effective methods for engaging different interest‐holders in evidence syntheses have not been identified [5]. Additionally, while there is some guidance related to engagement in research, it predominantly focuses on patient and public engagement in primary research, not evidence synthesis and there is limited guidance for engaging with other interest‐holders [3, 4, 7, 8, 9].

The aim of this paper is to introduce a series of articles about how to successfully engage different interest‐holders when conducting evidence syntheses. The series of articles will consider methods used to engage different interest‐holders (including who to involve and in what way), barriers and facilitators to engagement, impacts of engagement, management of conflicts of interest, and factors relating to equity.

This paper presents the shared definitions used across each of the five reviews included in this series. These reviews will inform the development of a guidance checklist and resources for engaging interest‐holders through all steps of evidence synthesis. The plan for developing this guidance is described in the project protocol [10].

“Interest‐holders” are groups of people with legitimate interests in the health issue under consideration and whose perspectives and views should be considered when conducting this study [2]. Their interests arise and draw their legitimacy from the fact that these people are responsible for or affected by health‐ and healthcare‐related decisions that can be informed by research evidence. Engagement of interest‐holders in evidence syntheses can promote transparency, accountability, trust, and help to ensure that the needs of interest‐holders are included. Engagement can improve the translation of evidence into policy and practice [11]. Interest‐holders can contribute throughout the steps of evidence synthesis including, for example, refining the research question and suggesting appropriate outcomes, suggesting additional references to consider, and providing context to interpret the evidence.

This study was conducted by the MuSE Consortium, a group of over 160 individuals from 20 countries who are interested in engagement in health research, evidence synthesis, and health guidelines. This project complements a previous MuSE project which developed guidance for engagement in the development of health guidelines and clinical practice recommendations [12].

## 2 Key Definitions

We have used a standardised set of terms and definitions that are used consistently across the set of reviews in this series. These definitions have been developed and agreed upon with the collaboration of the MuSE Consortium through our work related to engagement in research [6, 13, 14], guidelines [15, 16]; (Petkovic et al., 2022 [2, 10, 12, 17, 18, 19]), and in preparing the series of reviews being introduced here.

***Evidence syntheses*** synthesize the research evidence to address health care‐related questions. They use rigorous, explicit, and transparent methods and include scoping reviews, rapid reviews, and quantitative or qualitative systematic reviews [1]. There are a number of different types of evidence syntheses which are summarized in Table 1.

**Table 1:** Types of evidence syntheses.

Type of evidence synthesis	Description
Systematic Review (quantitative)	The term systematic review is commonly used in two different ways:
	a. as an umbrella term, meaning any form of evidence synthesis which employs rigorous methods. As such, the term systematic review is often used interchangeably with, or instead of, the term evidence synthesis. b. as a term to describe a quantitative evidence synthesis, for example “Cochrane systematic review” (to describe a review of randomised controlled trials, conducted using methods proposed by the Cochrane Collaboration).
	We use the latter, referring to a type of evidence synthesis which employs a rigorous methodology to synthesize data from all published studies on a specific topic or that answer a specific research question [20]. Systematic reviews are often used to assess the effectiveness of interventions based on the impact of their outcomes and typically involve an appraisal of the quality of available evidence [21]. Systematic reviews may also assess quantitative aspects such as diagnostic test accuracy, prevalence, prognosis and predictors of outcome. These evidence syntheses are used to inform decision making and may identify current gaps in research, identify areas for future research, and justify or contradict current clinical practice guidelines/conventions. The key characteristics of a systematic review are [22]:
	a clearly stated set of objectives with pre‐defined eligibility criteria for studies. an explicit, reproducible methodology. a systematic search that attempts to identify all studies that would meet the eligibility criteria. an assessment of the validity of the findings of the included studies, for example through the assessment of risk of bias; and systematic presentation, and synthesis, of the characteristics and findings of the included studies.
Qualitative Evidence Synthesis	Qualitative evidence syntheses, or qualitative systematic reviews, can focus on analyzing human experiences and cultural and social phenomena [23]. Like quantitative systematic reviews, qualitative reviews use rigorous methodologies, including clearly stated objectives and explicit reproducible methodologies. Using a qualitative approach to the systematic review process allows reviewers to synthesize rich data across methodologies to extract themes and deepen theoretical understanding. Qualitative evidence syntheses may be useful for investigating the experiential aspect of an intervention, typically when outcomes are measured with rich data (i.e., investigating patient experiences) [23, 24]. Other common terms to describe qualitative evidence syntheses include meta‐ethnography, meta‐narrative reviews, critical interpretive synthesis, and thematic synthesis [25].
Rapid Review	Rapid reviews, similar to systematic reviews, also synthesize the scope of data on a specific topic. However, rapid reviews are typically completed in a condensed fashion and so are opted for in the case of limited time and/or resources. They are typically less methodologically rigorous than typical systematic reviews but are useful for quickly summarizing data to adapt clinical practices [26].
Scoping Review	Scoping reviews are a type of evidence synthesis that aims to systematically identify and map the breadth of evidence available on a particular topic [27]. The information collected may summarize the volume of current research and its general themes and direction [20]. Scoping reviews can clarify key concepts/definitions in the literature and identify key characteristics or factors related to a concept, including those related to methodological research [27]. Scoping reviews may also be termed “mapping reviews” or may be used to populate Evidence and Gap Maps (EGMs) [27].
Realist Review	A realist review is a theory‐driven approach to reviewing the literature. The purpose of the review is to produce one or more theories to explain phenomena. Realist reviews aim to address questions about how, why, for whom, in what contexts and to what extent health systems, programmes and/or policies function. A realist perspective is based on the premise that for any observed outcome, there are one or more causal processes (called ‘mechanisms’) that only become active in certain contexts: Context (C) + Mechanism (M) = Outcome (O) [28]. A realist synthesis approach generally involves the engagement of interested people and groups as part of the method.
Mixed Methods Reviews	Mixed methods reviews refer to any combination of evidence synthesis methods, where one is typically a systematic review [21].
Overview of reviews	Overviews compile evidence from multiple systematic reviews into a single document, for example addressing a set of related interventions, diagnostic tests, populations, outcomes, or conditions. Overviews are aimed at decision makers, such as clinicians, policy makers, or informed consumers, and can address questions and sets of options that are often too broad for a single review [29]. Other terms used to describe overviews include “umbrella review” and “review of reviews.”
Living reviews	A living review is a systematic review which is continually updated, incorporating relevant new evidence as it becomes available. Practically, this means that living reviews [30]:
	Are underpinned by continual, active monitoring of the evidence (i.e. monthly searches) Immediately include any new important evidence (meaning data, studies or information) that is identified Are supported by up‐to‐date communication about the status of the review, and any new evidence being incorporated

***Interest‐holders*** include the following 11 P's: patients and caregivers, the public, providers of care, policy makers, program managers, payers of health research, payers of health services, peer review editors, and product makers. Table 2 provides a definition for each group. This taxonomy of interest‐holders groups is relevant to evidence synthesis and other taxonomies are used for other types of research, such as biomedical research, clinical research, and environment health research. A complete explanation of the term “interest‐holders” is provided in a commentary that is included within this series [2].

**Table 2:** Interest‐holders and their descriptions [17].

Broad categorization of interest‐holders	MuSE 11Ps	Description
Researchers	Principal Investigators and all members of the of research team	Individuals, organizations, and associations that conduct or advocate for health research, including the conduct of evidence syntheses
Patients and members of the public	Patients, patient caregivers, patient advocates/organizations	Those with lived experience of the condition of interest or who care for or advocate on behalf of those with lived experience
	Public	Individuals in the general population of a defined geographic area excluding patients, caregivers, and health professionals living or working with the condition of interest
Other professionals	Payers of health research	Individuals and organizations that fund research projects, such as government funders, industry funders, foundations
	Payers/Purchasers of Health Services	Individuals, organizations and entities that pay for health services
	Peer Reviewed Journals Editors	Those who set journal policy on guidelines and manage the peer review process and editing
	Policymakers	Individuals, organizations and entities that craft public or private policy on health
	Producers and commissioners	Institutions and organizations that commission, develop, or implement evidence syntheses
	Product makers	Individuals working for companies that manufacture pharmaceuticals, medical devices, medical procedures, health technologies, or for profit educational and behavioural packages
	Program managers	Managers/directors who plan, lead, oversee, or deliver any program that provides public health, community services, or clinical care (e.g. budgeting, hiring, staffing, organizing, coordinating, reporting)
	Providers	Persons and their professional associations who provide health care in a professional capacity or allowed by regulatory bodies to provide a health care service

*Engagement* refers to the two‐way relationship between interest‐holders and the research team. Other terms may be used, such as “involvement,” “partnership,” but for the purposes of our work we use “engagement” (Table 3).

**Table 3:** Terms related to engagement.

Term	Definition	Notes on use
Engagement	A bidirectional relationship, or collaboration between interested people and groups and a research team that results in informed decision‐making about the selection, conduct, and use of research [31, 32].	This is the preferred and most commonly used term in some parts of the world (including Canada and USA). It is therefore the term used in our series of evidence syntheses. It is important to note that the definition we use here is the same as the definition of the term involvement used in other parts of the world (e.g. UK). In the UK, and some other parts of the world, engagement is considered something distinct from involvement, generally comprising interactions which may fall short of the definition we provide here. However, within this series, we adopt the Canadian/US usage, and we do not make any distinction between engagement and involvement.
Involvement	Research being carried out “with” or “by” interested people or groups (rather than research carried out “about” or “for” interested people or groups).	To accommodate international variations in terminology, we use the same definitions for the terms engagement and involvement. At times we use the term involve in our work. For example, when referring to the questions within the ACTIVE framework (who was involved? etc) [33]. This reflects the original published terms.
Coproduction, and other terms starting with co‐ (e.g. co‐creation, codesign)	Internationally, there is no single, accepted definition of the term Coproduction, and it has been highlighted that anyone using this term should provide a definition [34]. Coproduction (and other co‐ terms) generally describe a process which adheres to a set of core values and principles. “Co‐” terms often refer to people working together in “a partnership” [8].	We consider these “co‐” terms specific approaches which are related to, but which may go beyond, engagement. Sharing of power and responsibility are key principles of these “co‐” terms [35] potentially creating fundamental differences from some approaches to engagement. As definitions vary, we will not use these terms in this study, instead describing the levels of engagement that interested people and groups may have.
Partnership	Like coproduction, partnership describes a specific approach in which particular values and principles are adhered to. It has been defined as “a relationship that involves mutual respect and an equal voice” [8].	As above, we consider partnership an approach which could go beyond minimum criteria for engagement. We will not use this term, but rather we will describe the levels of engagement that interested people and groups may have.
PPI (Patient and public involvement)	The term PPI refers to the active involvement of patients and the public in the research process [36]. Patients and the public are included within the interested people and groups relevant to this protocol (see below).	PPI is sometimes used as a noun, synonymous to involvement, sometimes referring to involvement of people and groups who are broader than “patients and public”. To ensure clarity we will avoid this abbreviation and describe the interested people and groups who are engaged using the terminology in Table 2.
Participatory research	An approach to research which values “participation from [interest‐holders] in the research process in specific ways to improve the quality and relevance of the research"to [37].	We will base our decision to include studies based on the description of engagement. We anticipate that participatory research will meet our definition for involvement of interested people and groups.

## 3 MuSE‐ES Series to Assess Engagement in Evidence Synthesis

This series comprises six papers addressing different aspects of engaging interest‐holders in evidence syntheses. These papers have each been co‐authored by a team that include representatives from a variety of our identified interest‐holder groups and all members of the MuSE Consortium were invited to contribute. Our first paper has already been published and introduces the term “interest‐holder [2].” The remaining five papers are evidence syntheses describing different issues related to engagement.

The first synthesis is a scoping review which identifies methods for engaging interest‐holders in evidence syntheses. This scoping review, is an update to a previous review [5], describes the methods used to engage interest‐holders, including who was engaged, the aims of their engagement, how they were engaged, and in what stages of the review process [38].

The second review is a mixed‐methods evidence synthesis examining the factors affecting the engagement of interest‐holders. Specifically, it aims to identify and synthesise what the barriers and facilitators are to involving interest‐holders across all stages of the review cycle using the Theoretical Domains Framework [39]. The review also examines how contextual factors may impact the nature and degree of involvement among different groups of interest‐holderst [40].

The third review assesses the impacts of interest‐holder engagement on evidence syntheses. For this review, “impact” with regard to engagement refers to “any change in the research process, the research product, the people involved, or broader society as a result of engagement in the conduct of an evidence synthesis” [41].

The fourth review describes issues related to conflicts of interest in engagement in evidence syntheses. This review identifies the types of conflicts of interest among different interest‐holders, how to manage them, and the impact of those conflicts on the evidence synthesis process [42].

Finally, the last review in the series aims to identify and characterize the equity considerations for the engagement of interest‐holders in evidence synthesis [10]. Equitable engagement focuses on the deliberate inclusion of diverse individuals and groups.

These reviews will inform the development of draft guidance for engagement with interest‐holders throughout the evidence synthesis process. We will explore agreement with this draft guidance through interviews with interest‐holders, and an international survey. We will use consensus methods to finalize a checklist for engaging with each of the 11 identified interest‐holder groups throughout the stages of the evidence synthesis process. We welcome expressions of interest to take part in the subsequent stages of this ongoing project.

## Author Contributions

**Jennifer Petkovic:** conceptualization, writing – original draft, project administration, writing – review and editing, funding acquisition. **Joanne Khabsa:** conceptualization, writing – original draft, writing – review and editing, funding acquisition. **Lyubov Lytvyn:** conceptualization, writing – original draft, writing – review and editing. **Alex Todhunter‐Brown:** conceptualization, writing – original draft, writing – review and editing, funding acquisition. **Olivia Magwood:** conceptualization, writing – review and editing, writing – original draft, funding acquisition. **Pauline Campbell:** conceptualization, writing – review and editing. **Elie A. Akl:** conceptualization, writing – review and editing, funding acquisition. **Thomas W. Concannon:** conceptualization, writing – review and editing, funding acquisition. **Holger Schunemann:** conceptualization, writing – review and editing, funding acquisition. **Vivian Welch:** conceptualization, writing – review and editing, funding acquisition. **Peter Tugwell:** conceptualization, supervision, writing – review and editing, funding acquisition.

## Disclosure

*Cochrane Evidence Synthesis and Methods*, as a Cochrane Collaboration journal abides by Cochrane's conflict of interest policy for Cochrane Library content (2020), which applies to all journal content. Cochrane's conflicts of interest policy for *Cochrane Evidence Synthesis and Methods* not only requires study funding and author interests to be declared at the earliest point possible, but also mandates that some funding and conflicts of interest will prevent people from being authors of submissions.

## Conflicts of Interest

The authors declare no conflicts of interest.

## Data Availability

Data sharing not applicable to this article as no datasets were generated or analysed during the current study.
